# Modified Mitral Valve Repair with Its Insufficiency of Ischemic Genesis

**DOI:** 10.17691/stm2021.13.2.07

**Published:** 2021-01-01

**Authors:** V.E. Vaykin, M.V. Ryazanov, D.D. Zhiltsov, S.A. Zhurko, A.B. Gamzaev, G.V. Bolshukhin, S.A. Fedorov, A.P. Medvedev

**Affiliations:** Cardiovascular Surgeon, Specialized Cardiosurgical Clinical Hospital named after Academician B.A. Korolev, 209 Vaneeva St., Nizhny Novgorod, 603136, Russia; Associate Professor, Department of Hospital Surgery named after B.A. Korolyov, Privolzhsky Research Medical University, 10/1 Minin and Pozharsky Square, Nizhny Novgorod, 603005, Russia; Cardiovascular Surgeon, Specialized Cardiosurgical Clinical Hospital named after Academician B.A. Korolev, 209 Vaneeva St., Nizhny Novgorod, 603136, Russia; Cardiovascular Surgeon, Specialized Cardiosurgical Clinical Hospital named after Academician B.A. Korolev, 209 Vaneeva St., Nizhny Novgorod, 603136, Russia; Professor, Department of X-ray Endovascular Diagnostics and Treatment, Privolzhsky Research Medical University, 10/1 Minin and Pozharsky Square, Nizhny Novgorod, 603005, Russia; Cardiovascular Surgeon, Specialized Cardiosurgical Clinical Hospital named after Academician B.A. Korolev, 209 Vaneeva St., Nizhny Novgorod, 603136, Russia; PhD Student, Department of Hospital Surgery named after B.A. Korolyov, Privolzhsky Research Medical University, 10/1 Minin and Pozharsky Square, Nizhny Novgorod, 603005, Russia; Cardiovascular Surgeon, Specialized Cardiosurgical Clinical Hospital named after Academician B.A. Korolev, 209 Vaneeva St., Nizhny Novgorod, 603136, Russia; Cardiovascular Surgeon, Specialized Cardiosurgical Clinical Hospital named after Academician B.A. Korolev, 209 Vaneeva St., Nizhny Novgorod, 603136, Russia; Professor, Department of Hospital Surgery named after B.A. Korolyov, Privolzhsky Research Medical University, 10/1 Minin and Pozharsky Square, Nizhny Novgorod, 603005, Russia

**Keywords:** ischemic mitral regurgitation, mitral valve reconstruction, coronary artery disease

## Abstract

**The aim of the study** was to assess the effectiveness of modified mitral valve repair in comparison with traditional methods of correcting ischemic mitral regurgitation.

**Materials and Methods:**

The results of surgical treatment of 80 patients with coronary artery disease complicated by ischemic mitral regurgitation were analyzed. The mean age of the patients was 58.95±8.36 years; the ratio of men and women was 67:13. Heart failure of FC II (according to the NYHA classification) was detected in 6 patients (7.50%), FC III — in 69 (86.25%) patients, FC IV — in 5 (6.25%) patients.

Echocardiographic examination was used to determine the significance and genesis of mitral regurgitation in the preoperative period. 57 patients (71.25%) were detected with grade II mitral regurgitation, 23 (28.75%) had grade III.

Annuloplasty was chosen as the operation for the correction of the valve apparatus. The patients of group 1 (n=23) underwent reconstructive surgery on the mitral valve using an autopericardial strip according to the technique, which we have developed, in combination with coronary artery bypass grafting (CABG), the patients of group 2 (n=26) underwent plastic surgery using a support ring in combination with CABG, patients of group 3 (n=31) had myocardial revascularization without correction of the valve apparatus.

**Results:**

The patients of group 2 underwent restrictive mitral annuloplasty performed with rigid support rings, group 1 — with an autopericardial strip as a soft support ring, the patients of group 3 underwent CABG alone.

One patient from group 2 died in the early postoperative period due to acute perioperative myocardial infarction.

The most common complications were pleurisy, acute cardiovascular failure, acute respiratory failure, and cardiac arrhythmias. The smallest number of complications was noted in the group 3, where patients underwent CABG alone. After surgery, all the patients showed a decrease in mitral regurgitation, which was most pronounced in the groups with annuloplasty.

When analyzing the immediate results of the operations, it was revealed that the patients of groups 1 and 2, who underwent combined interventions, had a higher percentage of complications, and the length of their stay in the ICU increased. However, these groups showed a significant improvement in mitral valve functioning. Plasty of the mitral valve with an autopericardial strip according to the technique, which we have developed, demonstrated a good hemodynamic effect, the absence of significant regurgitation in the postoperative period.

## Introduction

Nowadays, significant advances have been made in the surgical treatment of coronary artery disease, but the issue of choosing the best management tactics for patients with ischemic mitral regurgitation (IMR) is still controversial. In the North American and European guidelines for the treatment of patients with coronary artery disease with concomitant heart valve disease, there are no clear indications for the choice of the method of surgical treatment of IMR [1, 2]. It should be remembered that IMR is a disease of the left ventricular (LV) myocardium. The valve is usually unchanged, and regurgitation occurs due to changes in the architecture of the ventricle [3] and is quite dynamic: it often progresses on the mitral valve from minimal to severe within a short period of time. This depends mainly on the left ventricular load but is often associated with ongoing ischemia. Treatment of patients with IMR is difficult due to many complications caused by the progression of atherosclerosis. Besides, the traditional methods of mitral valve repair are rather limited: they work only in one plane and are not always able to solve problems with the subvalvular apparatus [1, 2].

**Figure 1:**
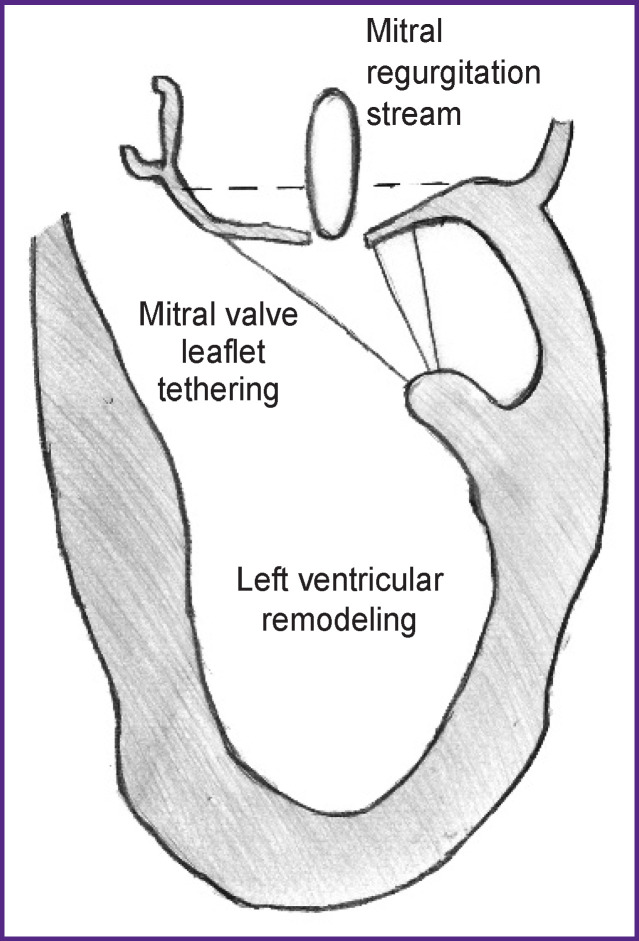
Mechanism of ischemic mitral insufficiency

Ischemic mitral regurgitation can be explained using the Carpentier classification of mitral regurgitation (MR) [4]. The etiology in this process is ischemic heart disease, and, by definition, patients should have a clinical picture of this pathological condition, a previous myocardial infarction (MI), a phenomenon of impaired local or global contractility, or dilatation (remodeling) of the LV. In IMR, leaflet tethering primarily changes as a result of ischemic ventricular remodeling. The posterolateral and apical displacement of the papillary muscles which correlates with the tethering distance [5, 6] leads to apical tethering, limitation of free margin mobility, and poor leaflet coaptation (Figure 1).

Secondary chordal tethering contributes to the seagull-shaped deformation of the anterior leaflet. Therefore, its correction is the goal of a certain group of plastic surgeries [7]. Though the both papillary muscles can be displaced, the dystopia of the posterior papillary muscle is usually more severe and results in a more aggressive tethering of the P3 region. This is particularly true in posterior MI associated with the posterior descending artery occlusion. In such cases, the stream of MR is usually eccentric, directed posteriorly along the P3 region [8, 9]. And, on the contrary, an infarction in the basin of the anterior descending artery leads to a global remodeling of the entire LV with the involvement of the both papillary muscles, tethering in all the regions of the leaflets, and the appearance of a central stream of regurgitation.

In contrast to primary mitral valve lesion, LV remodeling is often accompanied by dilatation and reshaping of the annulus, papillary muscle dystopia, and leaflet tethering. Annulus dilatation in patients with MI is less pronounced than in degenerative defects; therefore, restrictive annuloplasty is recommended in the case of leaflet tethering against the background of IMR. The posteromedial part of the annulus is more subject to changes [10–12], although, currently, the anterior part is also recognized to elongate and flatten out in the case of IMR. Posterior MI leads to septal-lateral dilatation along the A1–P3 axis, while anterior MI leads to more global septal-lateral dilatation, which causes symmetric and circular annulus enlargement [13].

Despite the fact that most patients with IMR have already had MI and LV remodeling, acute ischemia with impaired local contractility can lead to similar consequences. Under these conditions, revascularization alone contributes to the restoration of myocardial contractility and IMR disappearance. Elongation of the papillary muscles and prolapse of the valves are rare causes of IMR associated with MI, and usually do not require standing out of the more typical form of IMR caused by limited valve mobility. Isolated basal MI can lead to annular dilatation without tethering in the mitral leaflets, and this is, perhaps, the most favorable dysfunction associated with IMR [1, 2, 5].

**The aim of the study** was to assess the effectiveness of modified mitral valve repair in comparison with traditional methods of correcting ischemic mitral regurgitation.

## Materials and Methods

The retro- and prospective analysis of the case reports, the laboratory and instrumental findings of 80 patients with IMR who were treated at the Specialized Cardiosurgical Clinical Hospital named after Academician B.A. Korolev (Nizhny Novgorod, Russia) in 2015–2019 was carried out. The mean age of the patients was 58.95±8.36 years with the ratio of men and women of 67:13. Heart failure of II functional class (FC) according to the NYHA classification was detected in 6 patients (7.50%), III FC — in 69 (86.25%) patients, IV FC — in 5 (6.25%) patients.

The echocardiographic study was used to determine the significance and genesis of MR. In the preoperative period, grade II MR was observed in 57 patients (71.25%), grade III MR — in 23 (28.75%) patients. The grade of MR was assessed during transesophageal echocardiographic examination, which included the parameters describing the internal geometry of the mitral valve structures: the diameter of the annulus fibrosus, interpapillary distance, annulopapillary distance, leaflet tethering area, leaflet coaptation depth. There are several methods for the quantitative assessment of a tethering degree. The most common one is a simple measurement of the area from the leaflets to the plane of the mitral valve annulus which is usually performed into the middle of the systole when the area is at its minimum level. To determine the depth of coaptation, which correlates with the presence and severity of IMR, the maximum distance from the margin of the leaflets to the plane of the mitral valve annulus is measured [12].

All the patients were divided into 3 groups:

in group 1 (n=23), the reconstructive operations on the mitral valve were performed using an autopericardial strip as a soft support ring according to the technique we have developed, combined with coronary artery bypass grafting (CABG);

in group 2 (n=26), plastic surgery was performed using a rigid support ring in combination with CABG;

in group 3 (n=31), myocardial revascularization was performed without correction of the valve apparatus.

We have optimized the plasty procedure of the mitral valve with insufficiency of ischemic genesis using autopericardium fixed in a 0.6% solution of glutaraldehyde, as well as an original method for determining the required strip length. This made it possible to perform effective correction of the pathology without disturbing the anatomical configuration of the valve apparatus.

**Figure 2:**
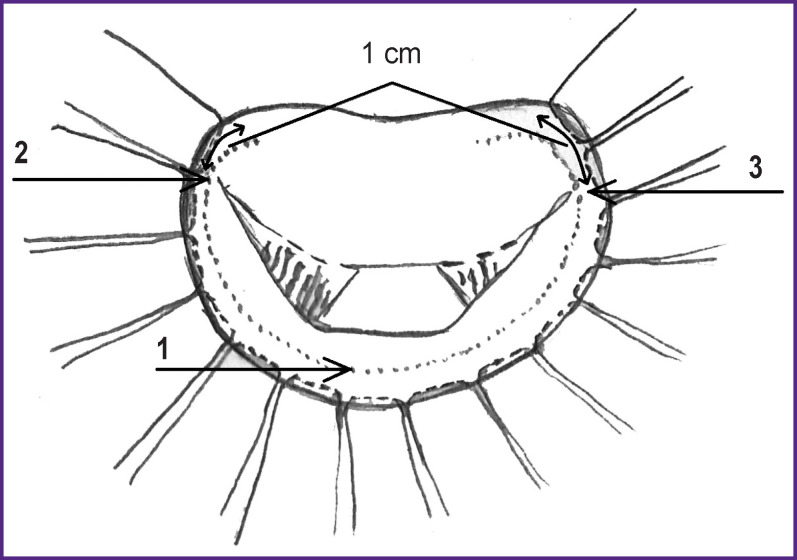
Mitral valve with U-shaped sutures along the base of the posterior leaflet (*1*) and 1 cm beyond the anterior (*2*) and posterior (*3*) commissures

At the stage of access to the heart, a strip 11–14 cm long and 6–7 mm wide was taken from the anterior surface of the pericardium, carefully cleaned from extraneous tissues, and immersed in a 0.6% glutaraldehyde solution for 10 min. Then it was washed alternately in three containers with a saline solution of 0.9% sodium chloride and stored until use. When exposed to glutaraldehyde, cross-links of collagen fibers are formed, which significantly reduce tissue bioresorption, increasing biocompatibility and atrombogenicity. Pericardium stabilized this way causes a minimal macrophage-lymphocytic reaction of the body, retains its histological structure, which allows for retaining its positive properties for a long time. After access to the mitral valve and its visualization, U-shaped sutures with braided lavsan threads covered with polybutyrate, size 2-0 according to USP, were applied onto the annulus fibrosus at the base of the posterior leaflet with 8–10 mm beyond the anterior and posterior commissures. The distance between the sutures was about 1 mm, the suture width was 5–7 mm (Figure 2).

**Figure 3:**
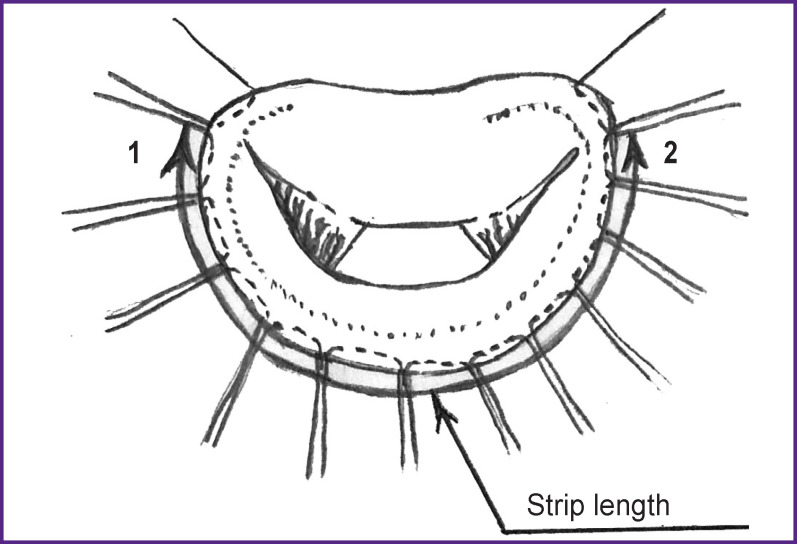
Measurement of the distance between the front (*1*) and back (*2*) commissures

After suturing, the distance between the commissures was measured along the length of the lower part of the annulus fibrosus with ligature (Figure 3). This measurement technique is determined by a greater susceptibility to changes in this particular part of the annulus fibrosus in IMR. The method of measurement that we have proposed allows for selecting the optimal length of the strip depending on the initial size of the fibrous ring of the mitral valve, achieving good coaptation of the leaflets, and, at the same time, preventing excessive overcorrection, that leads to an increase in the gradient on the mitral valve and occurrence of the SAM syndrome.

**Figure 4:**
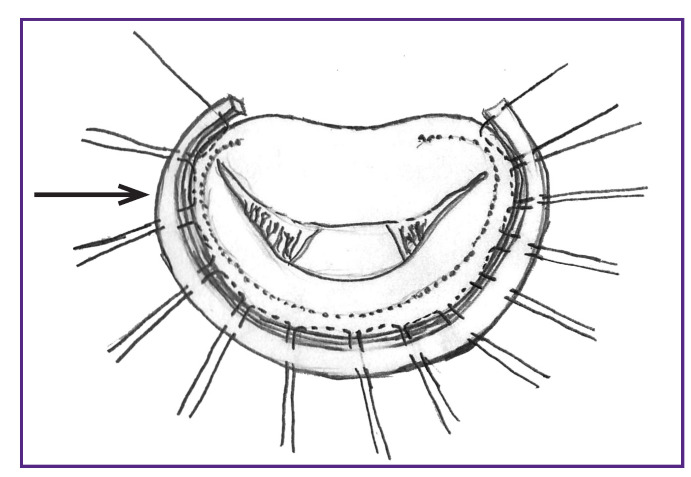
Stitched autopericardial strip (indicated by an arrow) is “planted” on the annulus fibrosus of the mitral valve

Then, a part of the autopericardial strip of the required (measured) length was cut away, fixed with two mosquito forceps so that the inner (serous) surface of the pericardium remained outward, and sutured with the threads used to suture the annulus fibrosus of the mitral valve (Figure 4).

**Figure 5:**
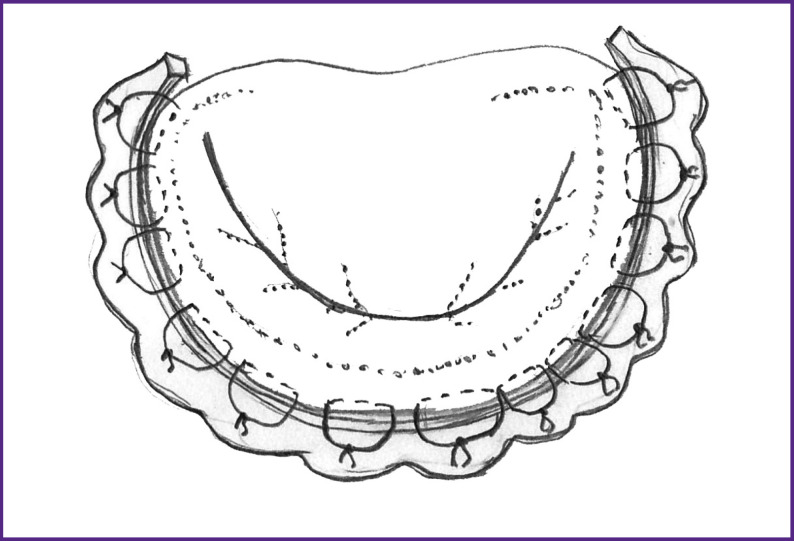
End result of plasty: all the sutures are tied, the annulus fibrosus of the mitral valve has been reduced, satisfactory leaflet coaptation has been achieved

After that, the strip was put onto the annulus fibrosus of the mitral valve, all the sutures were tied with five knots, and the threads were cut off (Figure 5).

Further, to confirm the consistency of the valve, a hydraulic test was performed, which consisted of filling the LV with fluid (0.9% sodium chloride solution) and visual observation of the presence of regurgitation on the valve. In case of its absence, the result of plasty was considered positive, the operation was completed. The grade of regurgitation was confirmed by transesophageal echocardiography. In the presence of marked regurgitation on the mitral valve, a decision was made on additional methods of correction, up to valve replacement.

**Statistical processing** was performed using the SPSS Statistica 19.0 software package. The presence of a statistically significant difference between the compared groups, represented by the numerical data in the corresponding samples that did not meet the normality criterion, was determined using the nonparametric Mann–Whitney test. The differences were considered statistically significant at p<0.05. The Kolmogorov–Smirnov method was used to assess the normal distribution of the samples of these parameters of the patients’ condition. For the samples that did not satisfy the criterion of normality, the data were presented as Me [Q1; Q3].

The presence of a statistically significant difference between the comparison groups, represented by the proportion (rate) manifestation of the desired property, was determined by the Fisher’s φ* method with angular transformation. The percentages were presented with indication of the percentage standard deviation (p±σp%).

The characteristics of the patient groups are presented in Table 1.

**Table 1:** Initial patient characteristics

Parameters	Group 1 — CABG + autopericardial strip (n=23)	Group 2 — CABG + ring (n=26)	Group 3 — CABG (n=31)	p
Age (years) (Me [Q1; Q3])	60.0	59.0	64.0	p_1–2_=0.452
	[55.0; 65.0]	[52.0; 63.0]	[59.0; 69.0]	**p_1–3_=0.011**
				**p_2–3_=0.002**
Ejection fraction (%)	50.0	49.5	52.0	p_1–2_=0.652
(Me [Q1; Q3])	[46.0; 55.0]	[45.8; 54.3]	[47.0; 58.0]	p_1–3_=0.362
				p_2–3_=0.180
End-diastolic volume (ml)	141.0	155.5	105.0	p_1–2_=0.009
(Me [Q1; Q3])	[131.0; 154.0]	[140.8; 174.3]	[94.0; 115.0]	**p_1–3_<0.001**
				**p_2–3_<0.001**
NYHA II (р±σ_р_):				p_1–2_>0.5
n	1	1	4	p_1–3_=0.11
%	4.35±4.25	3.85±3.77	12.90±6.02	p_2–3_=0.14
NYHA III (р±σ_р_):				p_1–2_>0.05
n	21	21	27	p_1–3_>0.5
%	91.30±5.88	80.77±7.73	87.10±6.02	p_2–3_>0.5
NYHA IV (р±σ_р_):				p_1–2_=0.091
n	1	4	0	p_1–3_=0.073
%	4.35±4.25	15.38±7.08	0.0	**p_2–3_<0.001**
Mitral regurgitation grade II+ (р±σ_р_):				p_1–2_>0.5
n	12	14	31	**p_1–3_<0.001**
%	52.17±10.42	53.85±9.78	100.0±0.0	**p_2–3_<0.001**
Mitral regurgitation grade III+ (р±σ_р_):				p_1–2_>0.5
n	11	12	0	**p_1–3_<0.001**
%	47.83±10.42	46.15±9.78	0.0	**p_2–3_<0.001**

## Results

26 patients underwent restrictive mitral annuloplasty with rigid support rings, 23 patients — with an autopericardial strip as a soft support ring. Besides, a significant part of the patients underwent plastic surgery of the tricuspid valve: 15 cases in group 1 (65.2%) and 13 cases in group 2 (50.0%). LV aneurysm repair was performed in 3 patients of group 1 (13.0%) and one in group 2 (3.8%). All the patients of group 3 underwent CABG alone. The echocardiography results and the results of the surgical interventions are presented in Table 2.

**Table 2:** Patient characteristics in the postoperative period (Me [Q1; Q3])

Parameters	Group 1 — CABG + autopericardial strip	Group 2 — CABG + ring	Group 3 — CABG	p
Myocardial revascularization index	3.0	3.0	3.0	p_1–2_=0.506
	[2.0; 4.0]	[2.0; 3.0]	[3.0; 4.0]	p_1–3_=0.703
				p_2–3_=0.248
End-diastolic volume (ml)	135.0	138.5	100.0	p_1–2_=0.078
	[115.0; 138.0]	[127.8; 142.5]	[90.0; 112.0]	**p_1–3_<0.001**
				**p_2–3_<0.001**
Ejection fraction (%)	52.0	50.0	52.0	p_1–2_=0.416
	[48.0; 56.0]	[45.5; 55.3]	[49.0; 56.0]	p_1–3_=0.661
				p_2–3_=0.172
ITU (bed-day)	3.0	3.0	2.0	p_1–2_=0.975
	[2.0; 5.0]	[2.0; 5.0]	[2.0; 3.0]	**p_1–3_=0.013**
				**p_2–3_=0.008**
Mitral regurgitation grade 0 (р±σ_р_):				p_1–2_>0.5
n	17	17	2	**p_1–3_<0.001**
%	73.91±9.16	65.38±9.33	6.45±4.41	**p_2–3_<0.001**
Mitral regurgitation grade I (р±σ_р_):				p_1–2_>0.5
n	6	9	23	**p_1–3_<0.001**
%	26.0±9.16	34.62±9.33	74.19±7.86	**p_2–3_<0.001**
Mitral regurgitation grade II (р±σ_р_):				p_1–2_>0.5
n	0	0	6	**p_1–3_<0.001**
%	0.0	0.0	19.35±7.10	**p_2–3_<0.001**

One patient from group 2 died in the early postoperative period because of acute perioperative MI. The most frequent complications were pleurisy, acute cardiovascular failure, acute respiratory failure, cardiac arrhythmias (Table 3). The least number of complications was noted in the group with CABG alone. After surgery, all the patients showed a reduction in MR, which was most pronounced in the groups with annuloplasty. LV ejection fraction increased in all the groups.

**Table 3:** Lethality and complications in the postoperative period (р±σ_р_)

Parameters	Group 1 — CABG + autopericardial strip (n=23)	Group 2 — CABG + ring (n=26)	Group 3 — CABG (n=31)	p
Lethality:				p_1–2_=0.062
n	1	0	0	p_1–3_=0.064
%	4.35±4.25	0.0	0.0	p_2–3_>0.5
Multiple organ dysfunction syndrome:				p_1–2_=0.2
n	3	1	1	p_1–3_=0.086
%	13.04±7.02	3.85±3.77	3.23±3.17	p_2–3_>0.5
Acute heart failure:				p_1–2_>0.5
n	2	4	1	p_1–3_>0.5
%	8.70±5.88	15.38±7.08	3.23±3.17	**p_2–3_=0.047**
Acute respiratory failure:				**p_1–2_=0.004**
n	3	0	2	p_1–3_>0.5
%	13.04±7.02	0.0	6.45±4.41	**p_2–3_=0.028**
Atrial fibrillation:				p_1–2_>0.5
n	1	2	2	p_1–3_>0.5
%	4.35±4.25	7.69±5.23	6.45±4.41	p_2–3_>0.5
Hemorrhage:				p_1–2_>0.5
n	1	2	0	p_1–3_=0.064
%	4.35±4.25	7.69±5.23	0.0	**p_2–3_=0.017**
Tamponade:				p_1–2_>0.5
n	1	2	0	p_1–3_=0.064
%	4.35±4.25	7.69±5.23	0.0	**p_2–3_=0.017**
Reosteosynthesis of the sternum:				p_1–2_>0.5
n	1	1	0	p_1–3_=0.064
%	4.35±4.25	3.85±3.77	0.0	p_2–3_=0.07
Encephalopathy:				p_1–2_>0.5
n	2	3	0	**p_1–3_=0.015**
%	8.70±5.88	11.54±6.27	0.0	**p_2–3_=0.003**
Pleurisy:				p_1–2_=0.3
n	4	8	1	**p_1–3_=0.035**
%	17.39±7.90	30.77±9.05	3.23±3.17	**p_2–3_<0.001**

## Discussion

Ischemic mitral regurgitation occurs due to ischemia or infarction of the LV posterolateral wall in the region of attachment of the posterior papillary muscle. LV dilatation and disfunction that cause lateral displacement of the posterior papillary muscle and limit the mobility of the posterior mitral leaflet, lead to secondary/functional regurgitation because of the inability of convergence of morphologically normal mitral leaflets (IIIb type dysfunction of the mitral valve by the Carpentier classification). In this case, CABG can improve this pathological situation if only the LV posterolateral wall is viable, ischemized and revascularization is possible. When the LV posterolateral wall is replaced by scar tissue, thinned, or having an aneurism, myocardial revascularization will hardly improve regional contractivity of the wall or change secondary MR. Also, if there is no target coronary artery in this area, then a significant improvement in the local contractility of the wall is unlikely after isolated CABG [14].

While correcting mitral valve in IMR, myocardium preservation and protection, and cardiopulmonary perfusion are significant. On exposure, the mitral valve may seem to be normal. The principal correction method is annulus reduction and, consequently, the reduction of the surface area of the valve. This hypercorrection technique was first proposed by Bolling in 1995 and since then has become a standard technical approach to IMR correction. A small (26^th^ or 28^th^) ring can be used in the majority of patients. Though initially there were concerns about probable problems related to systolic motion of the anterior leaflet and mitral stenosis due to hypercorrection, these mechanisms did not cause any unfavorable clinical complications in any long-term follow-up.

The type of ring used for mitral valve correction is considered important by many authors. Magne et al. [15] reported 80% of relapses in patients with grade II+ regurgitation 6 months after surgery for mitral valve plasty with large flexible and/or incomplete rings. Other researchers [16] have shown that correction with incomplete rings leads to high rates of MR recurrence, and residual IMR increases the likelihood of adverse events. Spoor et al. [17] reported a five-fold increase in MR relapses in patients with MV correction for IMR using flexible rings as opposed to closed rigid rings. Finally, Silberman et al. [18], when analyzing predictors of residual MR and recurrence of IMR, identified the LV size and ring type as the two most important factors influencing MR recurrence. According to the data [18], closed rigid rings showed the best results. On the other hand, according to our findings, the greatest changes occur in the posteromedial part of the annulus, which is precisely the main target for plasty with an autopericardial strip. In addition, the elastic structure of the strip does not interfere with the natural motion of the annulus fibrosus of the mitral valve during heartbeats. At the same time, it is possible to perform hypercorrection of local segments of the annulus fibrosus, where dilatation is more pronounced.

After the artificial circulation is ended, MR should not be detected by transesophageal echocardiography, either during or after surgery. Moreover, the coaptation area should be at least 8–10 mm (i.e. sufficiently deep). If this does not happen under anesthesia, then the risk of MR recurrence in the postoperative period increases. Also, the most important predictors of IMR relapse include the LV volume parameters (end-diastolic volume, end-systolic volume), interpapillary distance, papillo-annular distance, a restrictive type of LV diastolic dysfunction [19]. Patients with severe IMR, marked changes in LV architectonics, and the presence of risk factors for MR recurrence, should be thoroughly discussed as the possibilities of plastic surgery on the mitral valve in such cases are limited and, according to some studies [20], in a large number of cases they already become ineffective in the mid-term period.

## Conclusion

When analyzing the immediate results of the operations, it was revealed that the patients of groups 1 and 2 who had undergone combined interventions had a higher percentage of complications, a longer stay in the ICU. However, in these groups, there was a significant improvement in mitral valve functioning compared with the patients who had undergone coronary artery bypass grafting alone. Mitral valve plasty with an autopericardial strip according to the technique that we have developed demonstrated a good hemodynamic effect, the absence of significant regurgitation in the postoperative period.
